# Differences on Primary Care Labor Perceptions in Medical Students from 11 Latin American Countries

**DOI:** 10.1371/journal.pone.0159147

**Published:** 2016-07-14

**Authors:** Reneé Pereyra-Elías, Percy Mayta-Tristán, Juan José Montenegro-Idrogo, Christian R. Mejia, Gabriel Abudinén A., Rita Azucas-Peralta, Jorge Barrezueta-Fernandez, Luis Cerna-Urrutia, Adrián DaSilva-DeAbreu, Alvaro Mondragón-Cardona, Geovanna Moya, Christian D. Valverde-Solano, Rhanniel Theodorus-Villar, Maribel Vizárraga-León, Red-LIRHUS

**Affiliations:** 1 Escuela de Medicina, Universidad Peruana de Ciencias Aplicadas, Lima, Perú; 2 Facultad de Medicina, Universidad Nacional Mayor de San Marcos, Lima, Perú; 3 Hospital de Illapel, Illapel, Chile; 4 Facultad de Medicina, Universidad Nacional de Asunción, Asunción, Paraguay; 5 Facultad de Medicina, Universidad de Guayaquil, Guayaquil, Ecuador; 6 Facultad de Medicina, Universidad Dr. Jose Matias Delgado, San Salvador, El Salvador; 7 Facultad de Medicina, Universidad Central de Venezuela, Caracas, Venezuela; 8 Facultad de Medicina, Universidad Tecnológica de Pereira, Pereira, Colombia; 9 Facultad de Medicina, Universidad Nacional Autónoma de Honduras, Tegucigalpa, Honduras; 10 Facultad de Medicina, Universidad Hispanoamericana, San José, Costa Rica; 11 Facultad de Medicina, Universidad Católica Boliviana San Pablo, Santa Cruz de la Sierra, Bolivia; 12 Facultad de Medicina, Universidad Juárez, Durango, México; National Institute of Health, ITALY

## Abstract

**Background:**

The shortage in Latin-American Primary Care (PC) workforce may be due to negative perceptions about it. These perceptions might be probably influenced by particular features of health systems and academic environments, thus varying between countries.

**Methods:**

Observational, analytic and cross-sectional multicountry study that evaluated 9,561 first and fifth-year medical students from 63 medical schools of 11 Latin American countries through a survey. Perceptions on PC work was evaluated through a previously validated scale. Tertiles of the scores were created in order to compare the different countries. Crude and adjusted prevalence ratios were calculated using simple and multiple Poisson regression with robust variance.

**Results:**

Approximately 53% of subjects were female; mean age was 20.4±2.9 years; 35.5% were fifth-year students. Statistically significant differences were found between the study subjects’ country, using Peru as reference. Students from Chile, Colombia, Mexico and Paraguay perceived PC work more positively, while those from Ecuador showed a less favorable position. No differences were found among perceptions of Bolivian, Salvadoran, Honduran and Venezuelan students when compared to their Peruvian peers.

**Conclusions:**

Perceptions of PC among medical students from Latin America vary according to country. Considering such differences can be of major importance for potential local specific interventions.

## Introduction

Nearly 30 years after Alma Ata’s declaration for health systems reform through Primary Health Care (PHC), there are still sanitary disparities considered politically, socially and economically unacceptable [1]. Latin America is a region urging a health systems reorientation to PHC in order to provide the population with universal and equitable access to health [2,3].

To do so, it is necessary to reinforce Primary Care (PC)—defined as the first level of care, the “family doctor-patient scenario”, [4]—which is the cornerstone of PHC [1]. Theoretically, it constitutes the structure where integral and integrated health delivery from all health professionals is coordinated with the different levels of healthcare^1^. Besides improvement in infrastructure, strengthening of PC undoubtedly requires an increase in the availability of human resources for health (HRH). Currently, there is a shortage in skilled personnel terms [5,6]. Physicians in training constitute a key population in this aspect, because they will be the next generations of health workforce, which, appropriately oriented, could correct this crisis^5^.

Nevertheless, many reasons related to health systems, medical training and academic-professional expectations are described in such a way that enhance a negative perception of PC in doctors and medical students [7–11]. These factors, ultimately, are the reasons behind a future physicians’ choice not to work in this level of the health system [12,13].

In consequence, to intervene on PC medical workforce, it is especially necessary to identify perceptions about it. These factors may probably vary between countries, which might make recognizable potential ways of local intervention.

It is important to generate evidence in this matter [6] because Latin America has few reports to this date [14–16]. In the light of this context, the aim of our study is to evaluate the differences about PC labor perceptions in medical students from 11 Latin American countries.

## Materials and Methods

### Design and place of study

An observational, cross-sectional multicountry study was performed. It evaluated physicians in training from medical schools of Spanish-speaking Latin American countries. Eleven countries were included: Bolivia, Chile, Colombia, Costa Rica, Ecuador, El Salvador, Honduras, Mexico, Paraguay, Peru and Venezuela; with a total of 63 participating medical schools. Schools from Argentina, Cuba, Guatemala, Nicaragua, Panama and Uruguay were initially considered but declined to take part of the project [17].

### Study population

**Fig 1:**
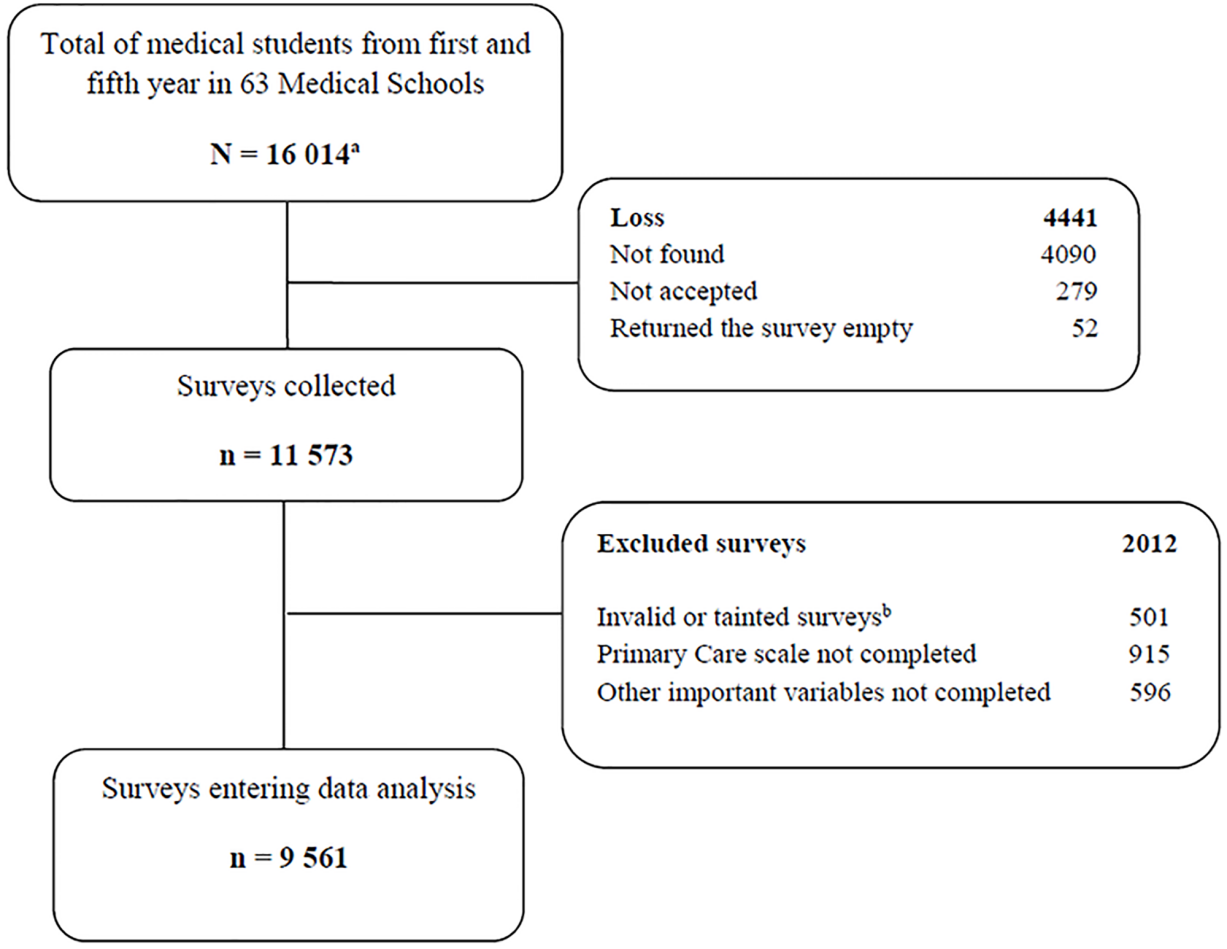
Flowchart of the study participants: Differences in Primary Care labor perceptions among medical students from 11 Latin American countries. ^a^ Total estimate of Medical Students from first and fifth year in participant Schools. ^b^ Surveys declared as invalidly or inappropriately fulfilled after revision.

We attempted to assess all medical students from the first and fifth year registered during the second semester of 2011 and the first semester of 2012 (approximately from September 2011 to July 2012). We included students that voluntarily accepted to complete the survey. We excluded those who returned tainted surveys and those with an unfinished or absent completion of the PC scale and/or other important variables (Fig 1).

### Procedures

Researchers were recruited through students associations’ networks, including a previously described Facebook strategy [18]. Eighty-six medical schools from 17 countries were initially participating; however, 23 schools eventually declined. At the end, 63 Schools from 11 countries were included. Details about study participants are described in Fig 1. All surveys were sent to the city of Lima, where the digitation process was carried out [17].

### Questionnaire and variables

The questionnaire was previously used in a pilot study where a Latin American students sample was assessed [19]. It was anonymous and self-administered. It evaluated sociodemographic aspects like sex (male vs female), age (in years), marital status (single vs others) and to be currently working for a payment. It also assessed variables related to family aspects, like having doctors as first-degree relatives (yes vs no), having children (yes vs no) and having at least one economic dependent person (yes vs no). Likewise, the questionnaire evaluated data related to the study subject’s academic profile, like year of study (first vs fifth), University funding (private vs public) and University location (capital vs out the capital), looking up to a physician working in a health center (yes vs no), English-language performance (advanced vs intermediate/basic) and performance on a native language different from Spanish (any vs none). It also comprised questions about the professional expectations, like emigration intention to work abroad, rural-setting labor intention, PC-facility labor intention, salary expectations (in US dollars) and the perception of the medical labor context in the country, specifically the salary (insufficient vs sufficient/more than sufficient). A translated version of the questionnaire is available (S1 File).

Perception about PC labor was measured through a previously validated scale in a Latin American students sample [20]. It comprises 11 items with a one-to-five Likert-type scale and evaluated the intensity of these perceptions. The total scores wide-ranged from 11 to 55 (simple summatory); higher scores reflect a negative perception of PC labor and, consequently, scores close to 11 imply positive perceptions. Additionally, this scale encompasses three differentiated domains: i) Perceptions about the PC physician (five items, 5–25 points), ii) Perceptions about PC labor itself (four items; 4–20 points) and iii) Perceptions about economic consequences of PC labor (two items; 2–10 points).

In this study, the internal consistency reliability analysis showed an adequate global Cronbach’s alpha: 0.84; ranging from 0.75 to 0.88 between countries. Cronbach’s alpha of the first domain (Factor 1) was 0.78 (0.66–078 according to each country) and the alpha of the second domain (Factor 2) was 0.74 (varying from 0.68 to 0.79). The third one (Factor 3) showed a lower internal consistency, α = 0.58 (0.46–0.65).

### Ethical issues

The original study was approved by the Ethics Committee of the Instituto Nacional de Salud (INS) from Peru. Furthermore, it was approved by the Research and/or Ethics Committees or competing authorities of medical schools were it was executed. This secondary data analysis was approved by the Ethics and Research Committee of the Universidad Peruana de Ciencias Aplicadas (Lima, Peru).

Before the survey was handed, students were informed about the study objectives and a verbal informed consent was obtained. When entering the study, we made sure their participation was voluntary and the survey anonymous.

### Data analysis

The database was generated with Microsoft Excel^®^ and, previous quality control, it was exported to STATA 11.0 (Stata Corp, Texas, USA). We used relative and absolute frequencies to describe categorical variables and means with standard deviations and medians with interquartile ranks (according to normality of the distribution) for numerical variables.

We generated tertiles for the overall PC perceptions scale score and for each-factor’s scores. For bivariate and multivariate analysis, we dichotomized this variable by separating those subjects with scores belonging to the first tertile (“favorable perceptions”) from the students with scores within the lower tertiles (“not favorable perceptions”). For bivariate analysis, we used the Student’s t-test when evaluating differences between the mean ages of those with favorable and not favorable perceptions. We used the χ^2^ (ji-square) for the analysis of categorical variables with the outcome.

We calculated crude and adjusted Prevalence Ratios (PR) with their respective 95% confidence intervals. For this, we used simple and multiple Poisson regression models with a robust error variance. We performed four different multivariate models with different levels of adjustment (sociodemographics, family aspects, academic profile and professional expectations—also considering medical schools as clusters) in order to ameliorate the influence of confounders and evidence the real effect of the country of origin. A P-value<0.05 was considered statistically significant.

## Results

### Study population

From the total population, we had response rates ranging from 59.6% to 100%, obtaining 11 563 surveys. From those, 9 561 were valid and entered data analysis (Fig 1). No differences were found between the final study subjects and the excluded group in terms of sociodemographics (p≥0.05).

52.9% were women and the mean age was 20.4±2.9 years. About 64.7% were first year students. Most of them were single (96.8%) and did not have children (96.6%). 64.0% studied in private schools. Only 9.2% had paid jobs and 7.6% had economic dependents. About 36.4% considered the national salary of the physician as insufficient. Characteristics of the study subjects are detailed in Table 1.

**Table 1:** Characteristics of Latin American medical students included according to their perceptions on Primary Care labor.

	Favorable		Not Favorable		Total	
	n	(%)	n	(%)	n	(%)
**Demographics**						
Male	1499	43.5	3006	49.2	4505	47.1^a^
Age^b^	20.4±3.0		20.4±2.8		20.4±2.9^c^	
Single	3358	97.4	5899	96.5	9257	96.8 ^a^
Paid job	281	8.2	598	9.8	879	9.2 ^a^
**Relatives**						
Physicians	1733	50.3	3036	49.7	4769	49.9
With children	130	3.8	286	4.7	416	4.4 ^a^
With economic dependents	192	5.6	531	8.7	723	7.6 ^a^
**Medical School**						
Fifth year	1125	32.6	2245	36.7	3370	35.3 ^a^
Private School	1290	37.4	2150	35.2	3440	36.0 ^a^
School in the capital city	1141	33.1	1889	30.9	3030	31.7 ^a^
Admires a family physician	256	7.4	359	5.9	615	6.4 ^a^
Advanced English performance	555	16.1	1125	18.4	1680	17.6 ^a^
Any native language performance	300	8.7	462	7.6	762	8.0 ^a^
**Perceptions on the national medical wages**						
More than sufficient	1366	39.6	2160	35.3	3526	36.9 ^a^
**Professional perspectives**						
Emigration	1095	31.8	2201	36.0	3296	34.5 ^a^
Rural setting	315	9.1	341	5.6	656	6.9 ^a^
Health center setting	181	5.3	242	4.0	423	4.4 ^a^
**Salary expectations**						
Not reported	1176	34.1	1583	25.9	2759	28.9^a^
<2000 US dollars a month	942	27.3	1661	27.2	2603	27.2
2000 to 5000 US dollars a month	805	23.3	1730	28.3	2535	26.5
>5000 US dollars a month	526	15.3	1138	18.6	1664	17.4

^a^ Statistically significant differences (chi^2^; p<0.05)

^b^ Mean and standard deviation.

^c^ No difference between means (Student’s T-test; p>0.05)

### Perceptions on PC labor

Scores of the scale were grouped in tertiles. Statistically significant differences were found between countries. Scores corresponding to the first tertile prevailed in countries like Chile (47.6%), Paraguay (47.3%) and México (44.9%). In Ecuador, however, the frequency of scores belonging to Tertile 3—suggesting a negative perception of PC labor—was considerably higher when compared to other countries (63.7%).

### Differences between perceptions on PC labor according to country: Multivariate model

In the multivariate model, differences were seen between countries, slightly variating among the different levels of adjustment for covariables (Table 2).

**Table 2:** Favorable perceptions on Primary Care: Multivariate models on differences between countries.

Country	Global score^a^	Tertile 1		Crude		Model 1^b^		Model 2^c^		Model 3^d^		Model 4^e^	
		n	(%)	PR	(95%CI)	PR	(95%CI)	PR	(95%CI)	PR	(95%CI)	PR	(95%CI)
Peru	33(9)	1189	(35.5)	1	-	1	-	1	-	1	-	1	-
Bolivia	33(9)	506	(37.0)	1.04	0.96–1.13	1.03	0.95–1.12	1.03	0.95–1.12	0.99	0.91–1.08	0.98	0.90–1.07
Chile	31(8)	273	(47.6)	1.34	1.22–1.48^f^	1.35	1.23–1.49^f^	1.35	1.22–1.49^f^	1.42	1.28–1.57^f^	1.33	1.19–1.48^f^
Colombia	32(9)	518	(40.6)	1.14	1.06–1.24 ^f^	1.14	1.05–1.23 ^f^	1.14	1.05–1.23 ^f^	1.15	1.06–1.26 ^f^	1.24	1.13–1.35 ^f^
Costa Rica	32(8)	56	(42.4)	1.19	0.97–1.46	1.18	0.96–1.44	1.18	0.96–1.44	1.15	0.93–1.43	1.10	0.89–1.36
Ecuador	39(13)	96	(11.9)	0.33	0.28–0.41 ^f^	0.34	0.28–0.41 ^f^	0.34	0.28–0.42 ^f^	0.33	0.27–0.41 ^f^	0.34	0.27–0.41 ^f^
El Salvador	32(8)	33	(37.5)	1.06	0.80–1.39	1.05	0.80–1.38	1.04	0.80–1.37	1.16	0.88–1.53	1.22	0.93–1.60
Honduras	32(9)	329	(39.5)	1.11	1.01–1.22 ^f^	1.11	1.01–1.22 ^f^	1.10	1.00–1.21 ^f^	1.12	0.99–1.25	1.07	0.95–1.20
Mexico	32(10)	83	(44.9)	1.26	1.07–1.49 ^f^	1.27	1.07–1.49 ^f^	1.26	1.07–1.49 ^f^	1.28	1.08–1.52 ^f^	1.26	1.06–1.50 ^f^
Paraguay	31(7)	69	(47.3)	1.33	1.11–1.59 ^f^	1.34	1.12–1.59 ^f^	1.34	1.12–1.59 ^f^	1.22	1.00–1.50 ^f^	1.27	1.03–1.55 ^f^
Venezuela	33(9)	297	(36.8)	1.04	0.94–1.15	1.02	0.92–1.12	1.01	0.92–1.12	1.01	0.91–1.13	1.07	0.95–1.20

^a^Median and interquartile range.

^b^Adjusted by sex, marital status and having a paid job.

^c^Adjusted by Model 1 + having a physician as relative and having an economically dependent person.

^d^Adjusted by Model 2 + year of study, going to a private school, going to a school located in the country’s capital, admiring a family physician, advanced performance on English language and any performance on a native language.

^e^ Adjusted by Model 3 + perception of the national medical wage, intention of emigration to labor abroad, rural-setting labor intention, intention to work in a health center facility and salary expectations.

^f^ Statistically significant differences (p<0.05)

In the complete multivariate model (Model 4—Table 2), students from Chile, Colombia, Mexico and Paraguay perceived PC labor more positively when compared to Peruvian students. We found no statistically significant differences with Bolivian, Costa Rican, Salvadorian, Honduran and Venezuelan students. Students from Ecuador showed less favorable perceptions than their Peruvian peers.

Table 3 details the differences between perceptions of students according to country by each one of the three factors the scale comprises. Only Chilean medical students considered PC favorable in all three dimensions of the scale when compared to Peruvian students. Likewise, being an Ecuadorean student was negatively associated with positive perceptions for all factors.

**Table 3:** Favorable perceptions of Latin American medical students on Primary Care (PC) labor: Differences between countries according to each factor of the PC labor perceptions’ scale.

Country	Factor 1 PC Phyisican			Factor 2 PC labor itself			Factor 3 Economic		
	%	PR^a^	(95%CI)	%	PR^a^	(CI95%)	%	PR^a^	(CI95%)
Peru	39.3	1	-	46.1	1	-	47.8	1	-
Bolivia	40.5	0.99	0.91–1.07	47.1	0.99	0.92–1.07	52.3	1.01	0.94–1.08
Chile	52.0	1.31	1.19–1.45^b^	52.7	1.20	1.09–1.32^b^	66.0	1.31	1.21–1.42^b^
Colombia	43.8	1.16	1.07–1.26^b^	57.7	1.31	1.23–1.40^b^	45.4	1.00	0.93–1.07
Costa Rica	50.0	1.23	1.03–1.48^b^	43.9	0.90	0.74–1.10	65.2	1.31	1.14–1.50^b^
Ecuador	12.9	0.33	0.28–0.41^b^	24.9	0.54	0.48–0.62^b^	31.7	0.64	0.57–0.72^b^
El Salvador	46.6	1.32	1.04–1.68^b^	52.3	1.15	0.94–1.41	37.5	1.00	0.77–1.30
Honduras	43.3	1.10	0.99–1.23	45.2	0.95	0.86–1.05	59.2	1.25	1.14–1.37^b^
Mexico	44.3	1.12	0.94–1.33	57.8	1.28	1.12–1.46^b^	55.1	1.11	0.96–1.28
Paraguay	47.3	1.18	0.97–1.44	63.7	1.28	1.10–1.48^b^	69.2	1.50	1.30–1.73^b^
Venezuela	46.3	1.20	1.09–1.33^b^	47.8	1.01	0.92–1.10	39.8	0.89	0.81–0.99^b^

^a^Adjusted by sex, marital status and having a paid job, having a physician as relative and having an economically dependent person, year of study, going to a private School, going to a School located in the country’s capital, admiring a family physician, advanced performance on English language and Any performance on a native language, perception of the national medical wage, intention of emigration to labor abroad, rural-setting labor intention, intention to work in a health center facility and salary expectations.

^b^Statistically significant differences (p<0.05).

## Discussion

Overall, results showed unfavorable perceptions about PC labor in the assessed medical students. Similar results have been previously reported elsewhere where the physicians shortage coexists with a remarked disinterest from the young workforce in this area [7,8,10,21]. In contrast, Zurro et al. found that a sample of more than five-thousand Spanish students from almost all medical schools in the country valued PC labor and the family physician very positively [22].

However, perceptions are far away from being similar between students included in our study. In the multivariate analysis, when adjusting by different sociodemographic variables and by professional expectations, differences persisted. These could be attributed to their own health systems’ characteristics, or singularities in local higher education.

We used Peru as reference because the census in the 33 existing Medical Schools (to the moment of the study) was accomplished and the number of study subjects accounts for approximately the third of the entire study population. The Peruvian health system also delineates an example of a developing regimen, fragmented, without universal coverage, without a defined long-term agenda and with strong internal inequities in terms of health access [23–26]. Moreover, it does not have a clear PHC orientation and does not consider PC a fundamental basis in practice [26]. In Peru, PHC is popularly misconceived as a precarious care service for those who cannot access for care in high-complexity facilities. These facilities are the ideal scenario where most patients wish to attend and most physicians aspire to work. In that sense, PC is seen as a less attractive labor option, especially in rural settings [23,27].

It is important to remark the differences found between subjects from the studied countries. Students from Chile, Colombia, Mexico and Paraguay perceived PC more positively in comparison to Peruvian students. These differences can be explained, mainly, because of the countries’ policies with respect of PC and PHC. As mentioned, particular characteristics of the health system can influence in the graduate and in training physicians’ perceptions about PC.

Chile and Colombia are examples of health systems that increased participation of the private sector, modernizing technologies and increasing efficiency [28–31]. In the case of Chile, for example, different strategies have been developed to strengthen PC in order to support PHC. The main axis of this reform was to increase PC workforce [32,33], raising 80% from 2004 to 2008 [34]. Besides, they launched initiatives to include PHC-oriented programs in medical schools [35]. This allowed the country to increase coverage and access, which notably elevated health indicators [36].

The health system of Paraguay is segmented, however since 2008 a PHC-centered national reform took off, with multidisciplinary teams that decentralize health care and increase access in their territory [37].

Students from Bolivia, Costa Rica El Salvador, Honduras and Venezuela might have similar perceptions to those from Peruvian students due to resemblances in their health systems or the role that PHC plays within them.

The case of Venezuela is particular. Since 2006, this country counts with the strategy “Misión Barrio Adentro”, which exhibits concentration on the integral community appraisal with PHC-skilled professionals as care deliverers [38]. In order to ensure and potentiate this scheme, they implemented a especial program to train family physicians [38,39]. Nevertheless, our results show that they have unfavorable perceptions about PC labor (comparable to Peruvian students’ perceptions). The qualitative exploratory study from Hernández & Gómez (2011) might contribute in the understanding of this phenomenon. They report to have found insecure social, economic and working conditions to be the main factor leading physicians to leave Venezuela [40]. It is also important to mention that the political conjuncture could someway explain part of the results [41]. Moreover, we did not evaluate students from this physician training program. These physicians may have different perceptions, which justifies later evaluation.

The health system of Costa Rica prioritizes PHC and displays a universal and solidary coverage. PC in this country holds multidisciplinary teams as the basic units of health delivery [42]. However, evaluated students do not have the positive perceptions expected. This can be due to the evaluation of a small sample belonging to only one university, thus it is not representative of the country.

On the other hand, Ecuadorean scores show much less favorable perceptions about PC. The arousal of a strong trend to empathize the secondary level-especially in rural areas- might enlighten our results’ meaning [43,44]. Furtherly, the reorientation of the Ecuadorean Health system to PHC took part very recently and thus, could not influence the study subjects' perceptions [45].

Even though we have analyzed cross-sectional data, our findings hold interesting possible implications. They suggest that, in countries where PC constitutes an important part of the health systems’ *dramatis personae*, students’ perceptions about PC are apparently more favorable and *vice versa*. Hence, we hypothesize that uplifting the role of PC in sanitary services and educational/academic settings might build a more positive regard for it within medical students and even licensed physicians.

We also present the scale factors individually. They allow us to appraise more disaggregated and clearly the students' perceptions about the PC physician, the PC labor itself and its economic reward. This way, specific contextual interventions can be planned. In that situation, stigmata related to PC labor can be identified and mitigated through education and motivation [10,46]. Likewise, factors that imply system deficiencies can be objective of health policies, like implementation of better wages and economic incentives [47] or an adequate PC-training orientation [48,49].

The study has some limitations. Characteristics of the subjects that refused to participate or were not found are unknown. We were not able to find information from the general population of the included schools, because this data was usually not available or not shared. Furthermore, a census was planned but it was not be achieved in most countries (all but Peru). This fact makes inference not possible to Latin America or even to the studied countries. However, this information could support later wider evaluations in more focalized settings when convenient.

To our knowledge, this is the largest continent-wide report evaluating the future workforce perceptions on PC, with almost ten thousand surveyed study subjects from sixty-three Medical Schools of eleven countries. This establishes it as a potential referent for the studied countries to understand students’ perceptions about PC labor and thereby intervene specifically.

We conclude that PC perceptions on PC vary according to the precedence country of the studied Latin American medical students, which might be due to contextual factors. We recommend the included nations to implement PHC-oriented specific normative-governmental and academic decisions. Thus, an environment where PC is well regarded in matters of political will, working conditions and also within the academic milieu, might be favorable for an eventual reframing of the medical community perceptions. This could make PC more attractive for the future workforce, in order to strengthen PC and, consequently, PHC, which is an indispensable philosophy to achieve universal health coverage.

## Supporting Information

S1 File Questionnaire of the Red-LIRHUS project. (PDF)
